# Retraction: A Human Torque Teno Virus Encodes a MicroRNA That Inhibits Interferon Signaling

**DOI:** 10.1371/journal.ppat.1009639

**Published:** 2021-06-01

**Authors:** The PLOS Pathogens Editors

After this article [1] was published, the corresponding author notified *PLOS Pathogens* that the microarray results and the findings reported in Table S3 and Figure 7E are not reliable.

The microarray data comprised one of two criteria used to identify candidate transcripts that are regulated by TTH8-miR-T1-miRNA. The original basis for identifying candidate transcripts included (i) reduced expression in the microarray analysis, and (ii), bioinformatic analysis demonstrating that the 3’ untranslated region (UTR) of the transcript contains a seed match to TTH8-miR-T1miRNA. A review of the original data from the microarray commercial source suggested that the microarray sample labels were not correctly represented in the dataset, and as such the microarray results reported in [1] are not reliable. These data, which underlie Table S3 in [1] and were deposited in the GEO repository as dataset GSE51520, should be disregarded in light of this issue. The authors have requested removal of this dataset from the GEO repository.

While the microarray results must be discounted, the authors noted that the results reported in Figure 6 provide support for the claim that the antiviral gene, N-myc (and STAT) interactor (NMI), is a target of TTH8-miR-T1miRNA and that TTV-tth8-miR-T1 or NMI depletion can inhibit interferon signaling (Figures 7B and C).

Importantly, however, the original reagents are no longer available for the B-cell growth assay that was used to generate Figure 7E. This result had formed part of the basis for the conclusion of reduced interferon response associated with TTH8-miR-T1miRNA in cells. In an attempt to reproduce the original reported work, the authors regenerated similar lentiviral reagents for replication experiments but were unable to establish stable cell lines in the GM19240 B-cell background. The LCL cells used for the experiments shown in Figure 7B appear to be refractory to efficient lentiviral transduction. As the authors were unable to replicate the findings reported in Figure 7E, they concluded that these data, which formed the foundation of the original interferon-suppression-of-growth assay, are not reliable.

Members of the *PLOS Pathogens* Editorial Board evaluated the impact of the microarray and Figure 7E results on the article’s overall results and conclusions. The editors concluded that the microarray data should be deleted from the public record but would not in isolation be critical to the article’s main results. However, the data in Figure 7E played a key role in verifying that expression of the TTV-tth8 miRNA serves to provide some protection from the antiviral effects of interferon. Without such biological validation data, the editors concluded that key aspects of the article’s results and conclusions are not adequately supported, and the article should be retracted.

The authors noted that they are unable to pursue further biological validation experiments because there is currently no *in vivo* or cell culture model of TTV infection.

In light of the above issues and the outcome of the editorial assessment, the *PLOS Pathogens* Editors retract this article.

CSS apologizes for the issues with the published article and regrets that they were not identified prior to publication. JMB and EdV likewise apologize for the issues. RPK, CSS, and JMB stand by the validity of the article’s results, except for the microarray data and Figure 7E results.

RPK, JMB, EdV, and CSS agreed with retraction. JCC either could not be reached or did not respond directly to comment on this decision.
